# Association between cardiac time intervals and incident heart failure after acute coronary syndrome

**DOI:** 10.1007/s10554-024-03206-8

**Published:** 2024-08-03

**Authors:** Caroline Løkke Bjerregaard, Flemming Javier Olsen, Kristoffer Grundtvig Skaarup, Peter Godsk Jørgensen, Søren Galatius, Sune Pedersen, Allan Iversen, Tor Biering-Sørensen

**Affiliations:** 1 Department of Cardiology, Copenhagen University Hospital - Herlev and Gentofte, Hellerup, Denmark; 2 Cardiovascular Non-Invasive Imaging Research Laboratory, Department of Cardiology, Herlev & Gentofte Hospital, University of Copenhagen, Niels Andersens Vej 65, 2900 Hellerup, Denmark; 3 Department of Biomedical Sciences, Center for Translational Cardiology and Pragmatic Randomized Trials, Faculty of Health and Medical Sciences, University of Copenhagen, Copenhagen, Denmark; 4 Department of Cardiology, Copenhagen University Hospital – Bispebjerg and Frederiksberg, Copenhagen, Denmark; 5 Department of Biomedical Sciences, Faculty of Health and Medical Sciences, University of Copenhagen, Copenhagen, Denmark; 6 Department of Cardiology, Copenhagen University Hospital – Rigshospitalet, Copenhagen, Denmark

**Keywords:** Cardiac time intervals, Heart failure, Acute coronary syndrome, Event timing

## Abstract

**Background:**

Cardiac time intervals are sensitive markers of myocardial dysfunction that predispose to heart failure (HF). We aimed to investigate the association between cardiac time intervals and HF in patients with acute coronary syndrome (ACS).

**Methods:**

This study included 386 ACS patients treated with percutaneous coronary intervention (PCI). Patients underwent an echocardiography examination a median of two days after PCI. Cardiac time intervals including isovolumic relaxation time (IVRT), isovolumic contraction time (IVCT), and systolic ejection time (ET), and myocardial performance index (MPI) were obtained by tissue Doppler echocardiography. The outcome was incident HF.

**Results:**

During follow-up (median 4.3, IQR:1.0-6.7 years), 140 (36%) developed HF. In unadjusted analyses, IVRT was not associated with HF (HR 1.02 (0.95–1.10), *p* = 0.61, per 10ms increase), and neither was IVCT (HR 0.07 (0.95–1.22), *p* = 0.26, per 10ms increase). Increasing MPI was associated with a higher risk of HF (HR 1.20 (1.08–1.34), *P* = 0.001, per 0.1 increase), and so was decreasing ET (HR 1.13 (1.07–1.18), *P* < 0.001 per 10ms decrease). After multivariable adjustment for cardiovascular risk factors, MPI (HR 1.13 (1.01–1.27), *P* = 0.034) and ET (HR 1.09 (1.01–1.17), *P* = 0.025) remained significantly associated with incident HF. LVEF modified the association between ET and HF (p for interaction = 0.002), such that ET was associated with HF in patients with LVEF ≥ 36% (HR = 1.15 (1.06–1.24), *P* = 0.001, per 10ms decrease).

**Conclusion:**

In patients admitted with ACS, shortened ET and higher MPI were independently associated with an increased risk of incident HF. Additionally, ET was associated with incident HF in patients with LVEF above 36%.

**Graphical Abstract:**

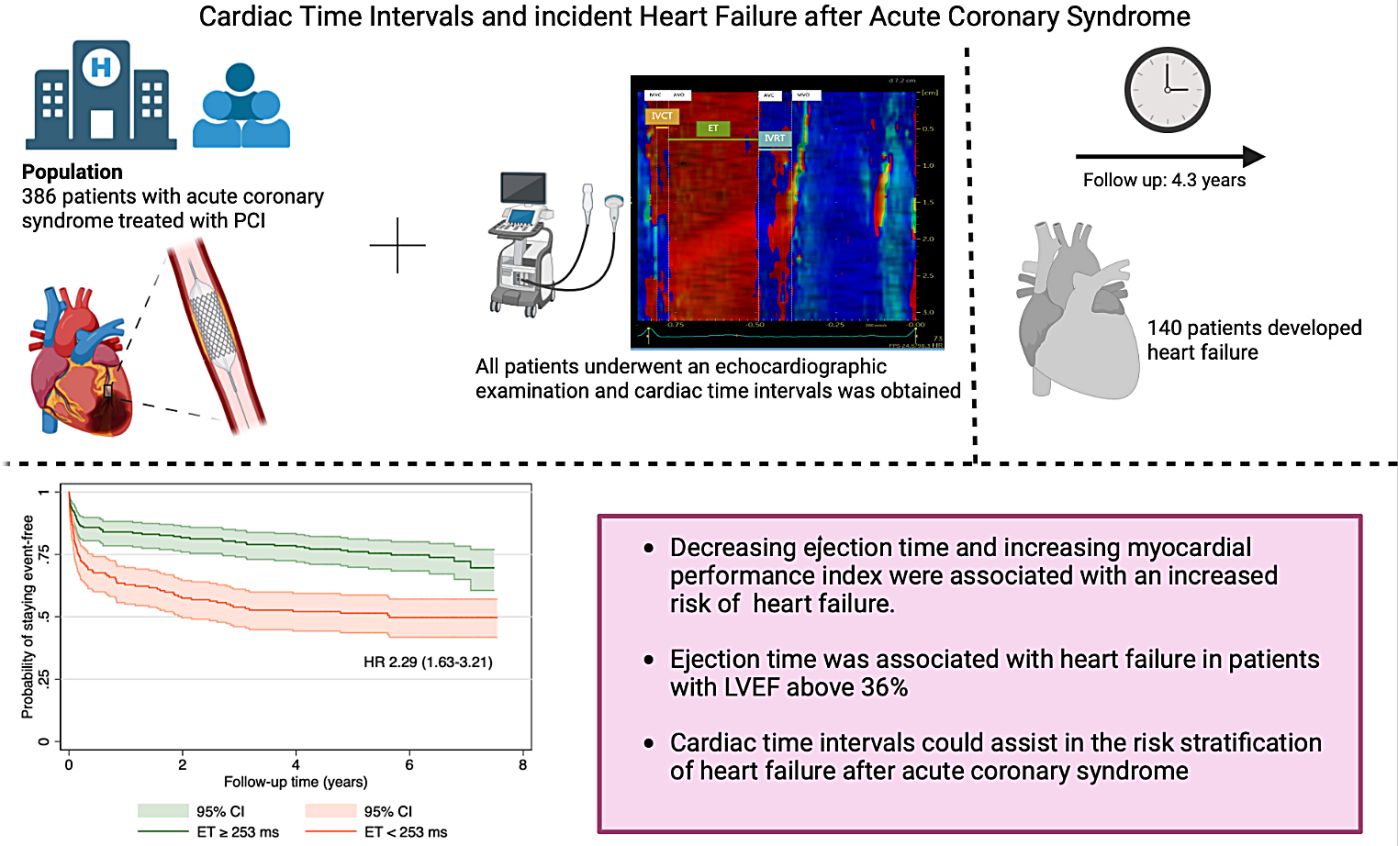

**Supplementary Information:**

The online version contains supplementary material available at 10.1007/s10554-024-03206-8.

## Introduction

Acute coronary syndrome (ACS) frequently leads to impairment of left ventricular (LV) function and is consequently the major cause of heart failure (HF) [1, 2]. Both systolic and diastolic LV dysfunction can develop prior to symptom onset, caused by structural and/or functional cardiac abnormalities, which are precursors for HF [3–5]. Consequently, identifying sensitive markers in patients at risk of HF is pivotal to institute preventive measures and guide timely initiation of guideline-directed medical therapy once patients exhibit signs of HF. This can potentially reduce morbidity and mortality following ischemic events [6].

Currently, the main focus and echocardiographic risk marker for HF after ACS is the LV ejection fraction (LVEF) [7]. However, LVEF is a measure of systolic function and does not provide insight as to diastolic dysfunction [8]. Furthermore, it represents a crude measure of systolic function and is generally perceived as a late-stage finding in the progression of systolic dysfunction [9, 10] Within recent years, a novel Doppler method – the color tissue Doppler imaging curved M-mode – has emerged as a reproducible and accurate method for estimating cardiac time intervals. Cardiac time intervals encompass isovolumic phases (isovolumic relaxation and contraction time), systolic ejection time (ET), and the myocardial performance index (MPI) as a combined measure of systolic and diastolic dysfunction [11, 12]. Preservation of normal cardiac time intervals is closely associated with normal cardiac mechanics [13] and will change in the ailing heart and worsen with disease progression. Accordingly, they may identify minor impairments in cardiac function, which are normally not recognized by conventional echocardiography While cardiac time intervals have shown potential value in several conditions of ischemic heart disease [11, 14], it remains to be explored in a broader group of patients with ACS. Therefore, the aim of this study was to investigate the association between cardiac time intervals and HF in patients with ACS treated with percutaneous coronary intervention (PCI).

## Methods

### Study population

This was a retrospective cohort study of 5,003 patients treated with PCI at the Department of Cardiology, Herlev and Gentofte Hospital, Copenhagen University, Denmark from January 2003 through November 2008. We hereby identified 580 ACS patients defined as one of the following: ST-segment elevation myocardial infarction (STEMI), non-ST-segment elevation myocardial infarction (NSTEMI), or unstable angina pectoris (UAP).

All patients underwent a detailed echocardiography examination performed at a median of 2 days (IQR: 1–3 days) post-PCI procedure. For patients with NSTEMI or UAP, the median time the index event to PCI was 1 day (IQR: 0–2 days). In total 115 patients were excluded due to non-sinus rhythm, missing images inadequate image quality for acquisition of conventional echocardiographic measurements. In addition, patients in whom the cardiac time intervals were not measurable were excluded (n: 73) and so were patients with a history of HF (n: 22). Finally, 386 patients were left for final analysis. The study population has previously been described in detail. [15, 16] In compliance with Danish regulations, retrospective studies such as this one do not require approval from an ethics committee.

### Echocardiography

Echocardiography was performed using GE Vivid ultrasound systems (GE Healthcare, Little Chalfont, UK) by experienced clinicians and sonographers. The images were stored and analyzed offline by one experienced investigator blinded to clinical baseline data and endpoints. Analysis was done using EchoPac version 113 (GE Healthcare, Horten, Norway).

### Conventional 2D echocardiography

Chamber quantifications were performed according to guidelines [17]. Interventricular septal width at end-diastole (IVSd), left ventricular posterior wall width at end-diastole (LVPWd), left ventricular internal diameter at end-diastole (LVIDd), were all measured from the parasternal long axis view at the tip of the mitral leaflet. LV mass index (LVMI) was calculated by LV mass divided by body surface area.

From the mitral valve inflow patterns using pulsed-wave Doppler imaging at the tip of the mitral valve leaflets of the 4-chamber view early filling (E), late filling (A), as well as the E/A-ratio and the deceleration time (DT) of the E-wave was assessed. Pulsed-wave tissue Doppler imaging from the lateral and septal annulus was used to measure peak longitudinal early diastolic tissue velocity (e’). The E-wave velocity was indexed to e´ to obtain E/e’.

LVEF was measured by the Simpsons biplane method. Left atrial volume (LAV) was measured by the biplane area-length method.

### Cardiac time intervals

**Fig. 1:**
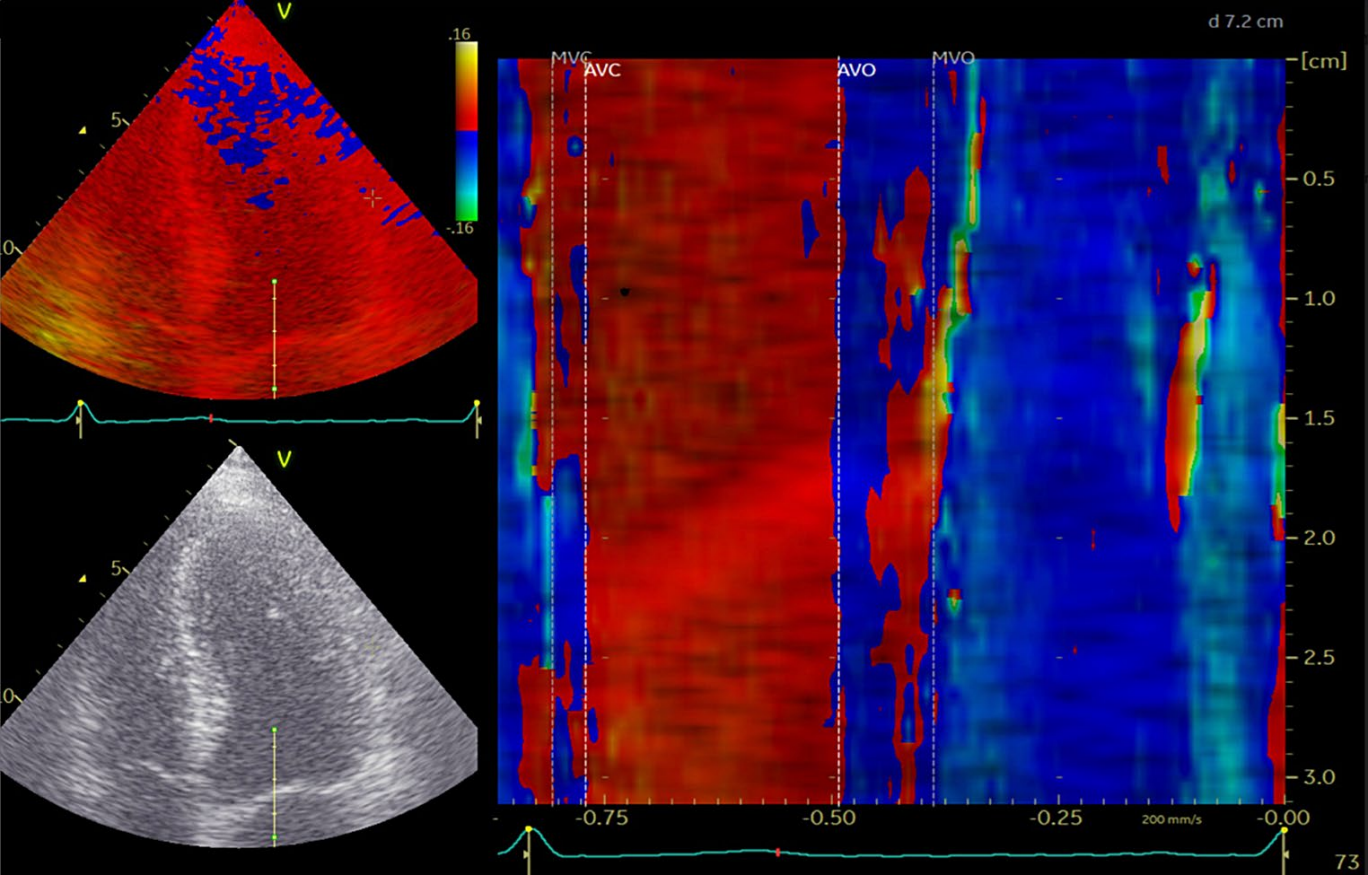
Cardiac time interval measurement. The cardiac time intervals assessed by color tissue Doppler imaging (TDI) M-mode through the mitral leaflet. *Abbreviations* AVO; aortic valve opening, AVC; aortic valve closing, MVC; mitral valve closing, MVO; mitral valve opening

Valvular event timing was measured by color tissue Doppler imaging M-mode by placing a 1–2 cm curved M-mode line through the anterior mitral valve leaflet in the apical 4-chamber view to obtain a color-coded spectrum of the valve motion throughout the cardiac cycle (Fig. 1). This color-coded spectrum provides direct estimates of mitral valve closure (MVC) and opening (MVO), which is defined as the development of aliasing phenomena as the valve motion exceeds the Nyquist limit. Consequently, MVC is defined as the color shift turquoise-red-blue and MVO is defined as the shift in color from red to yellow. Aortic valve opening (AVO) and closure is not measured directly, but indirectly as the traction on the mitral valve leaflet from left ventricular motion that occurs with aortic valve opening and closure. Consequently, AVO is defined as the color shift from blue to red, and AVC as the color shift from red to blue.

The cardiac time intervals were calculated from these valvular event timings. The isovolumic contraction time (IVCT) was defined as the time from MVC to AVO. The ET was defined as the time from the AVO to AVC. The isovolumic relaxation time (IVRT) was defined as the time from AVC to MVO. The MPI was calculated as the sum of the two isovolumic periods divided by ET ([IVRT + IVCT]/ET). The method has previously been described and validated [11, 14, 18].

### Outcome

The endpoint was incident HF hospitalization. The endpoint was obtained from the Danish National Board of Health’s National Patient Registry using the International Classification of Diseases-10 diagnostic codes (ICD-10). We defined incident HF as ICD-10 code I50.

### Statistics

Gaussians distribution of continuous variables were assessed by histograms with those showing normal distribution presented as mean ± standard deviation and compared with the Student’s T-test, whereas non-Gaussians-distributed variables were expressed as median values with interquartile ranges (IQR) and compared with the Mann-Whitney U test. Categorical variables are presented as frequencies and compared with the Chi2-test. Patients were compared according to HF outcome status, according to whether patients were included vs. excluded in the study, and by comparing patients with high vs. low values of ET (cut-off 253 ms). Cut-off values for high vs. low values of IVRT, IVCT, ET, and MPI were chosen based on area under the curve (AUC) from receiver operating characteristics curves. These AUCs were compared to AUCs obtained from conventional measures of diastolic function (E/A, e’, E/e’). Increment in AUC for the cardiac time intervals compared to standard diastolic measures (E/e’ and e’) were made with DeLong’s method.

Time-to-first-event analyses were performed by Cox proportional hazards regression. Proportional hazards were evaluated by Schoenfeld residuals. Linearity was tested through restricted cubic spline curves (see below). We evaluated whether LVEF modified the association between cardiac time intervals and outcome with a test for interaction. Since LVEF modified the association between ET and HF, we determined the optimal LVEF cut-off for effect-modification by testing for interaction per 1% increment in LVEF with simultaneous assessment of AIC. Optimal LVEF cut-off was determined to 36%. Accordingly, the association between ET and HF was also investigated in patients stratified according to this cut-off.

Multivariable adjustments were made in sequential steps as follows:

Model 1 was adjusted for age, gender, body mass index, systolic blood pressure, diastolic blood pressure, diabetes, current smoker, hypercholesteremia, heart rate, and family history of CVD. Model 2 was adjusted for the same variables as model 1 and by diagnosis (STEMI vs. UAP/NSTEMI), multivessel disease, and LAD culprit lesion.

Restricted cubic spline curves based on Poisson regression were constructed to depict the continuous association between the cardiac time intervals and HF. The number of knots were chosen based on the association which yielded the lowest Akaike Information Criterion (AIC).

Kaplan-Meier curves were constructed and depicted by high vs. low values of cardiac time intervals based on median values.

All statistical analyses were performed with STATA Statistics/Data analysis, SE 15.1 (StataCorp, Texas, USA). A p-value < 0.05 was considered significant in all tests.

## Results

### Baseline clinical characteristics

Baseline clinical, angiographic, and echocardiographic characteristics of the study population and stratified by low vs. high ET are presented in Table 1.

**Table 1:** Baseline characteristics for the entire study population and stratified by high vs. low systolic ejection time

Variable	All	Low ET (< 253 ms)	High ET (≥ 253 ms)	*P*-value
	(*n* = 386)	(*n* = 167)	(*n* = 219)	
**Clinical characteristics**				
Age, years	64 ± 12	64 ± 12	64 ± 12	0.74
Male gender, n (%)	283 (73)	125 (75)	158 (72)	0.55
Heart rate, beats per minute	73 ± 14	81 ± 14	67 ± 10	< 0.001
Body mass index, kg/m^2^	26 ± 4	26 ± 5	27 ± 4	0.25
Systolic blood pressure, mmHg	138 ± 25	136 ± 27	140 ± 24	0.16
Diastolic blood pressure, mmHg	82 ± 16	82 ± 17	82 ± 15	0.85
Mean arterial pressure, mmHg	119 ± 21	118 ± 23	121 ± 19	0.24
Diabetes mellitus, n (%)	34 (9)	14 (8)	20 (9)	0.80
Current smokers, n (%)	184 (48)	86 (52)	98 (45)	0.19
Hypercholesterolemia, n (%)	88 (23)	34 (20)	54 (25)	0.32
Family history of CV disease, n (%)	119 (31)	52 (31)	67 (31)	0.91
Prior CV disease, n (%)	32 (8)	10 (6)	22 (10)	0.15
**Hospitalization**				
*Diagnosis*				
STEMI, n (%)	294 (76)	139 (83)	155 (71)	0.004
NSTEMI and/or UAP, n (%)	92 (24)	28 (17)	64 (29)	0.004
*Culprit lesion*				< 0.001
Left circumflex artery, n (%)	52 (14)	13 (8)	39 (18)	
Left anterior descending artery, n (%)	195 (51)	112 (67)	83 (38)	
Right coronary artery, n (%)	139 (36)	42 (25)	97 (44)	
Multivessel disease, n (%)	23 (6)	8 (5)	15 (7)	0.40
**Echocardiography**				
Interventricular septal thickness, cm	1.1 ± 0.2	1.1 ± 0.2	1.0 ± 0.2	0.37
LV internal diameter, cm	4.9 ± 0.6	4.9 ± 0.6	4.9 ± 0.5	0.54
LV posterior wall thickness, cm	1.0 ± 0.2	1.0 ± 0.2	1.0 ± 0.2	0.23
Left ventricular mass index, g/m^2^	96 ± 23	98 ± 27	94 ± 18	0.28
Left ventricular ejection fraction, %	42 ± 11	37 ± 11	45 ± 9	< 0.001
E/A ratio	0.98 [0.79–1.23]	0.91 [0.78–1.19]	1.01 [0.81–1.26]	0.033
E/e’ ratio	9.7 [7.9–12.4]	10.0 [8.0-13.2]	9.3 [7.7–12.0]	0.055
e’, cm/s	7.4 ± 2.3	6.8 ± 2.2	7.8 ± 2.3	< 0.001
Deceleration time, ms	173 ± 46	164 ± 50	179 ± 42	< 0.001
Left atrial volume index, mL/m^2^	29.4 ± 10.7	29.6 ± 11.9	29.3 ± 9.7	0.91
TAPSE, cm	1.9 ± 0.4	1.8 ± 0.4	2.0 ± 0.4	< 0.001
Global longitudinal strain, %	-13.1 ± 3.7	-11.1 ± 3.5	-14.6 ± 3.1	< 0.001
*Cardiac time intervals*				
IVRT, ms	99 ± 24	100 ± 27	99 ± 21	0.59
IVCT, ms	27 [18–37)]	26 [17–38]	28 [20–37]	0.19
ET, ms	259 [237–281]	234 [218–245]	278 [266–293]	< 0.001
MPI	0.49 [0.41–0.57]	0.55 [0.46–0.65]	0.46 [0.38–0.52]	< 0.001

Continuous variables with Gaussian distribution are shown as mean ± standard deviations, skewed variables as median with interquartile range, and proportion as total numbers and percentages

*Abbreviations* CV: Cardiovascular; LV: Left ventricular; STEMI: ST-elevation myocardial infarction; NSTEMI: Non-ST-elevation myocardial infarction; UAP: Unstable angina pectoris; TAPSE: Tricuspid annular plane systolic excursion; IVRT: Isovolumic relaxation time; IVCT: Isovolumic contraction time, ET: systolic ejection time; MPI: Myocardial performance index

The mean age was 64 ± 12 years, 73% were men, and 76% presented with a STEMI. Patients with lower ET had a higher heart rate (81 vs. 67 beats per minute), and more frequently had STEMI (83% vs. 71%) and left anterior descending (LAD) lesions (67% vs. 38%). They presented with worse diastolic function by the e’ (6.8 vs. 7.8 cm/s) and E/A ratio (0.91 vs. 1.01) and deceleration time (164 vs. 179 ms) and RV systolic function, as well as significantly reduced systolic function by LVEF (37% vs. 45%) and GLS (-11.1% vs. -14.6%). Patients who were included were significantly younger and had lower heart rate, but higher blood pressure and more were active smokers compared to those excluded from this analysis. In addition, no patient who were included had left main lesion, whereas 3% of those who were excluded had left main lesion (Supplementary Table 1).

### Association between cardiac time intervals and heart failure

Of the 386 patients included in the study, 140 (36%) developed HF during a median follow-up of 4.3 (IQR: 1.0-6.7) years. Supplementary Table 2 details clinical, angiographic, and echocardiographic characteristics stratified by the outcome.

Those who developed HF during follow-up had a significantly higher heart rate (77 vs. 71 beats per minute), more frequently had prior cardiovascular disease (13% vs. 6%), and more frequently had LAD lesions as the culprit lesion (63% vs. 44%). They exhibited significantly worse LV systolic function (LVEF: 37% vs. 44%; GLS: -11.0% vs. -14.4%), diastolic function, and slightly lower RV systolic function.

By cardiac time intervals, no between-group differences were noted for IVRT or IVCT. However, those who developed HF exhibited significantly shorter ET (248 vs. 267 ms, *p* < 0.001) and higher MPI (0.53 vs. 0.48, *p* = 0.007).

The associations between the cardiac time intervals and incident HF by Cox proportional hazards regression are shown in Table 2. IVRT and IVCT were not associated with outcome, but decreasing ET and higher MPI posed a significantly increased risk of HF (HR 1.13 (1.07–1.28), per 10 ms decrease in ET and 1.20 (1.08–1.34), per 0.1 increase in MPI). Optimal cut-off values from ROC-curves were determined to be 98 ms for IVRT (AUC 0.52, 95% CI: 0.47–0.58), 25 ms for IVCT (AUC 0.51, 95% CI: 0.44–0.58), 253 ms for ET (AUC 0.65, 95% CI: 0.60–0.71), 0.54 for MPI (AUC 0.58, 95% CI: 0.52–0.64). By comparison, a lower AUC was observed for the E/A, with an AUC of 0.46 (95% CI: 0.40–0.53) with an optimal cut-off of 0.9, whereas the AUCs for the E/e’ and e’ were comparable to the cardiac time intervals. The AUC was 0.58 (95% CI: 0.51–0.64) for E/e’ (cut-off of 11.6), and 0.62 (95% CI: 0.56–0.68) for e’ (cut-off of 6.9 cm/s).

**Table 2:** Cox regressions for cardiac time intervals

	HR (95% CI)	*P*-value
Univariable		
IVRT, per 10 ms increase	1.02 (0.95–1.10)	0.61
IVCT, per 10 ms increase	1.07 (0.95–1.22)	0.26
ET, per 10 ms decrease	1.13 (1.07–1.18)	< 0.001
MPI, per 0.1 increase	1.20 (1.08–1.34)	0.001
Model 1		
IVRT, per 10 ms increase	1.05 (0.98–1.13)	0.17
IVCT, per 10 ms increase	1.12 (0.99–1.27)	0.07
ET, per 10 ms decrease	1.12 (1.04–1.20)	0.002
MPI, per 0.1 increase	1.16 (1.04–1.30)	0.008
Model 2		
IVRT, per 10 ms increase	1.04 (0.96–1.12)	0.32
IVCT, per 10 ms increase	1.13 (1.00-1.29)	0.050
ET, per 10 ms decrease	1.09 (1.01–1.17)	0.025
MPI, per 0.1 increase	1.13 (1.01–1.27)	0.034

Model 1 is adjusted for age, gender, body mass index, systolic blood pressure, diastolic blood pressure, diabetes, current smoker, hypercholesteremia, heart rate, and family history of CVD.

Model 2 is adjusted for model 1, STEMI, multivessel disease, and LAD.

*Abbreviations* IVRT: Isovolumic relaxation time; IVCT: Isovolumic contraction time, ET: systolic ejection time

**Fig. 2:**
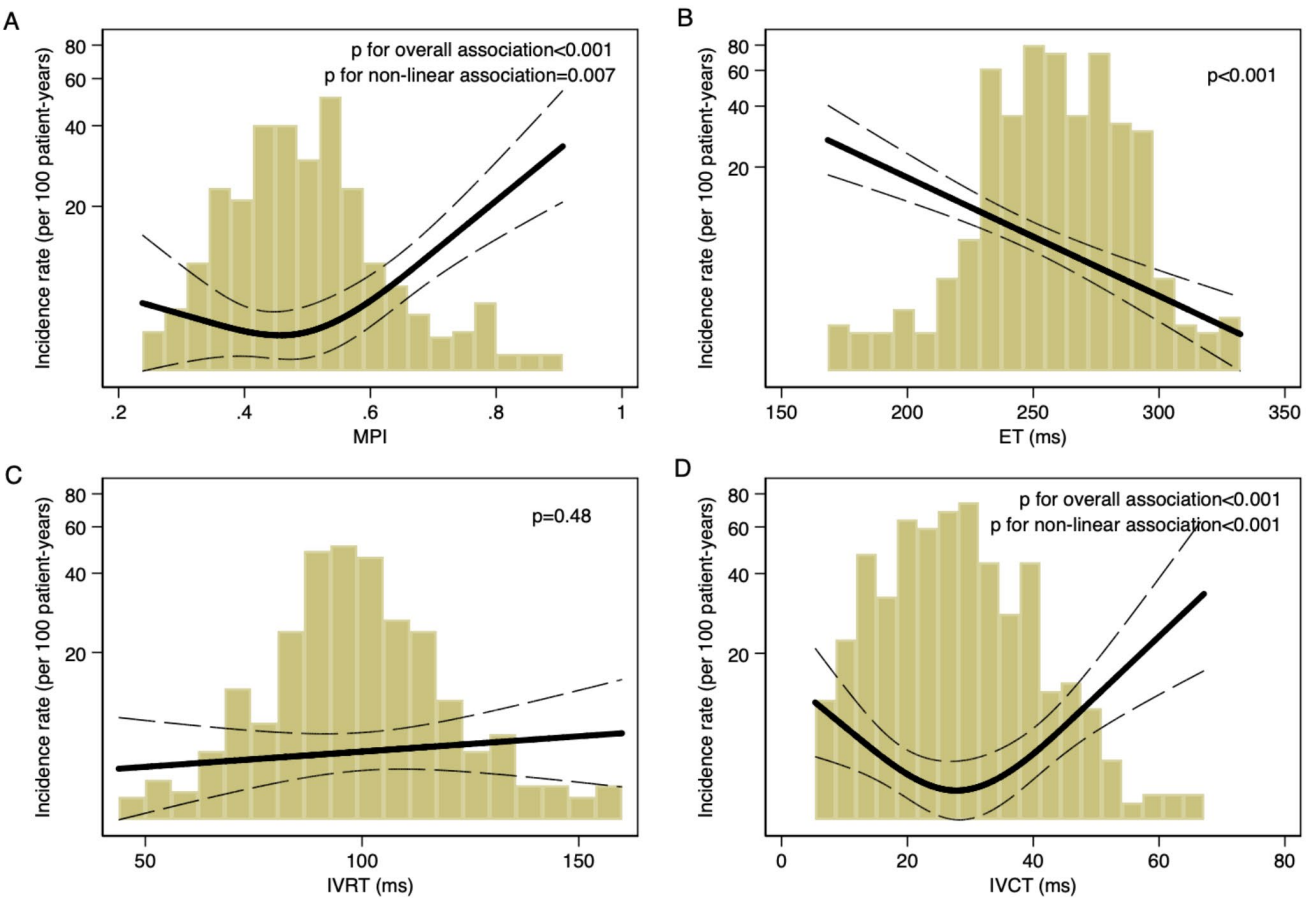
Association between cardiac time intervals and heart failure The figure shows a restricted cubic spline curve, illustrating the relationship between cardiac time intervals (x-axis) and the incidence of heart failure per 100 patient years (y-axis). The underlying histograms are also shown with proportions of patients with different values. Solid line represents the unadjusted incidence rate and the dotted lines represent 95% CI. *Abbreviations* IVRT; isovolumic relaxation time, IVCT; isovolumic contraction time, ET; systolic ejection time

The continuous association between these cardiac time intervals and HF is shown in Fig. 2a-d. Of note, MPI and IVCT showed a curvilinear association with the outcome, whereas a linear association was observed for ET and the outcome.

After adjusting for cardiovascular risk factors (model 1), only MPI and ET were associated with HF (Table 2), and similar findings were noted after more extended adjustments (model 2), by which IVCT also showed a trend towards being associated with HF.

**Fig. 3:**
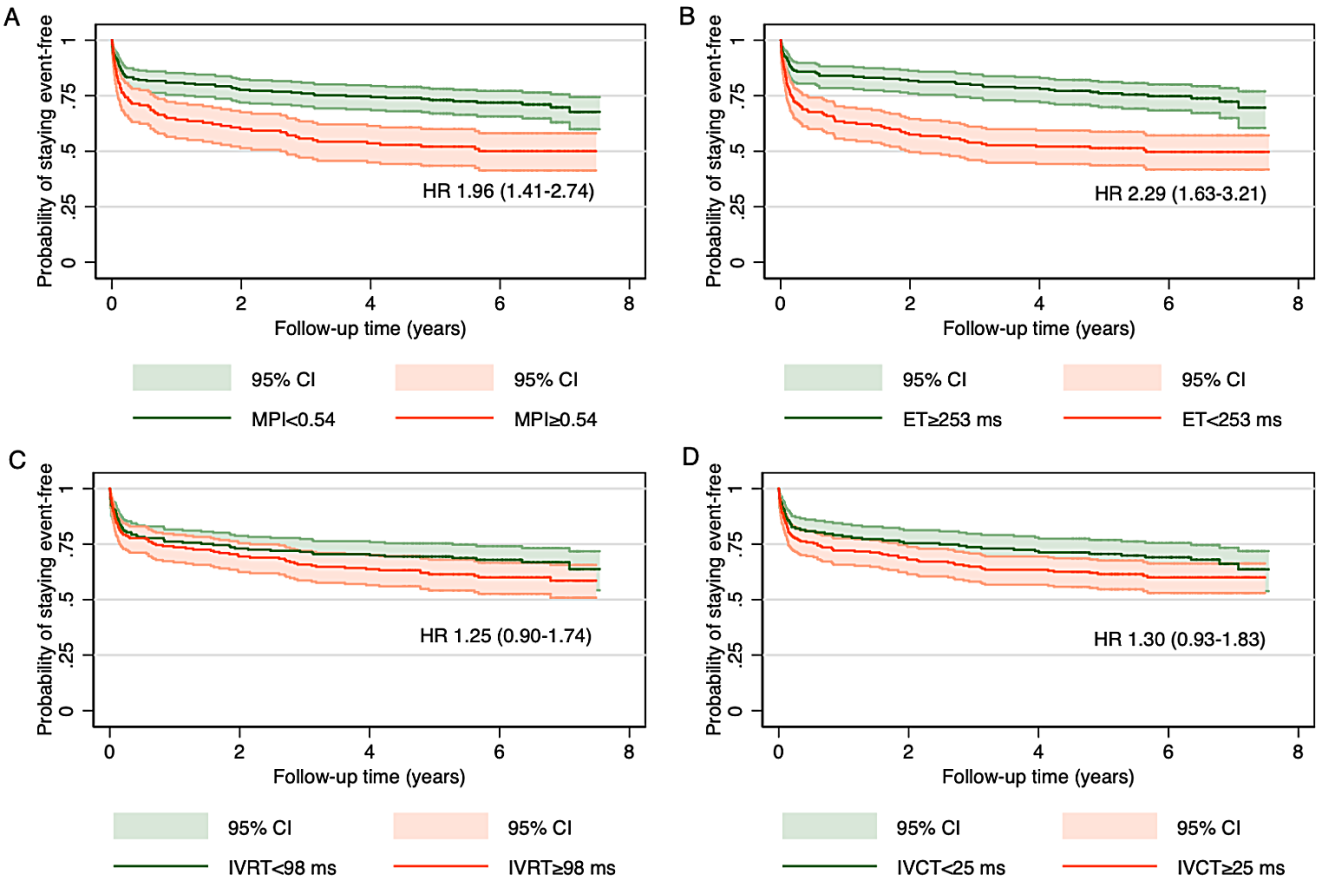
Kaplan Meier curves according to cardiac time intervals and heart failure. Kaplan Meier estimates, showing the probability of staying event free for patients stratified into stratified by high vs. low values of **(A)** myocardial performance index **(B)** systolic ejection time **(C)** isovolumic relaxation time, and **(D)** isovolumic contraction time. *Abbreviations* IVRT; isovolumic relaxation time, IVCT; isovolumic contraction time, ET; systolic ejection time, HR; hazard ratio

When the population was stratified into high vs. low values of cardiac time intervals, we observed that a high MPI posed a 96% increased risk of HF (HR 1.96 (1.41–2.74) (Fig. 3a), low ET posed a more than two-fold increased risk of HF (HR 2.29 (1.63–3.21) (Fig. 3b), whereas high IVRT and high IVCT were not associated with an increased risk of HF (Fig. 3c-d).

MPI did not yield significantly higher AUC when added to E/e’ nor e’ (p for increment in AUC = 0.79 and 0.39, respectively). ET did yield significantly higher AUC when added to E/e’ (p for increment in AUC = 0.040) but not when added to e’ (p for increment in AUC = 0.27).

### Cardiac time intervals with preserved ejection fraction

LVEF significantly modified the association between ET and outcome (p for interaction = 0.002) but did not modify the association between the other cardiac time intervals and outcome (p for interaction > 0.05).

ET was not associated with HF in patients with LVEF below 36% (n: 116, events: 70) (HR 1.01 (0.94–1.08), per 10 ms decrease, *p* = 0.78), whereas in patients with LVEF ≥ 36% (n: 270, events: 70), ET was associated with HF (HR 1.15 (1.06–1.24), per 10 ms decrease, *p* = 0.001). This association persisted after multivariable adjustments (HR 1.13 (1.02–1.26), per 10 ms decrease, *p* = 0.023). However, the association did not persist after more extended multivariable adjustments in model 2 (HR 1.09 (0.98–1.21), per 10 ms decrease, *p* = 0.13).

No association between the other cardiac time intervals and HF was observed in this subgroup of patients with LVEF ≥ 36% (IVRT: HR 0.96 (0.86–1.07), per 10 ms increase, *p* = 0.48; IVCT: HR 0.97 (0.80–1.18), per 10 ms increase, *p* = 0.79; MPI: HR 1.10 (0.91–1.33), per 0.1 increase, *p* = 0.32).

## Discussion

In the present study, we investigated the association between cardiac time intervals obtained from color TDI M-mode and the risk of developing HF in patients with ACS. The key findings were that shortened ET and higher values of MPI posed an increased risk for the development of HF after multivariable adjustments. In addition, shortening of the ET was associated with an increased risk of HF in patients with relatively preserved systolic function by LVEF.

While we revealed independent associations between both ET and MPI to incident HF, it is important to note that the AUCs obtained by both cardiac time intervals and diastolic measures were of modest value. In addition, we did not observe that any of the cardiac time intervals yielded incremental prognostic information compared to standard measures of diastolic function, in particular the e’, albeit ET did significantly increase the AUC compared to E/e’.

### Doppler method for estimating cardiac time intervals

Throughout decades, cardiac time intervals have been investigated as potential markers of cardiac dysfunction [19–22] but a main concern has been how to achieve these intervals in an easy, timely, non-invasive, and reproducible manner.

Originally, the cardiac time intervals were assessed through velocity curves derived from spectral Doppler. This method was introduced by Tei et al. [21, 22], but has certain limitations, including the need for at least two projections and consequently two cardiac cycles, making it prone to heart rate variability in-between the two projections. [23, 24]. As a consequence, another non-invasive method to obtain cardiac time intervals was proposed by tracking mitral valve movement in color tissue Doppler curved M-mode [25–28]. By direct comparison, this method found that the cardiac time intervals were associated with invasive LV pressure measurements of systolic function, which was not the case by the spectral Doppler method [29]. In addition, the curved M-mode method is obtained from one projection and one cardiac cycle, thereby eliminating beat-to-beat variation. It has also been shown to have better precision and reproducibility compared with the conventional method described by Tei and colleagues [23, 30]. Even when good imaging quality is difficult to obtain, it is often possible to visualize the mitral valve in the apical projection, which makes it possible to assess the cardiac time intervals even with suboptimal image quality and may therefore be of advantage in clinical settings.

### Cardiac time intervals across the heart failure spectrum

How cardiac time intervals are associated with HF has been described across a broad spectrum of populations. Based on the Copenhagen City Heart Study, Alhakak et al. reported on the value of cardiac time intervals for predicting HF in 1892 participants from the general population. Of these 1892 participants, 172 (9%) HF events occurred during 16 (IQR:12–16) years of follow-up, and the authors also found IVCT and MPI to be significantly associated with HF after multivariable adjustments [31]. Interestingly, they did not find ET to be significantly associated with HF after multivariable adjustments. This discrepancy with our findings may rely on the difference in the populations only a small fraction of the participants in the Copenhagen City Heart Study had ischemic heart disease (5%).

This is supported by findings from patients with suspected chronic coronary syndrome, in whom Olsen et al. have previously shown that ET, IVRT/ET, and MPI were independently associated with high calcium score and coronary artery stenosis by cardiac CT in patients with suspected coronary artery disease and seemingly normal echocardiograms [14]. The same pattern has been observed in patients with overt ischemic heart disease. This includes a study of 391 STEMI patients in which high IVRT/ET and MPI were found to be associated with HF [11]. However, after extensive multivariable adjustment none of the cardiac time intervals were associated with HF – nor any other individual endpoint - but only with a combined endpoint of new MI, hospitalization with HF, and all-cause mortality, which could be a matter of few events for the individual endpoints, whereas in the current study the number of HF events were markedly higher.

In patients with HF, ET also seems to provide important clinical information. It has previously been demonstrated that ET was significantly shortened and IVCT significantly prolonged in patients with mild-to-moderate HF [32]. In addition, ET has been reported to be independently associated with an increased risk of cardiovascular morbidity and mortality in patients with HF with reduced EF [33]. A more recent study on patients with reduced LVEF found decreasing ET to be associated with established systolic and diastolic function measurements, and ET remained significantly associated with all-cause death [34]. In our study, we found that decreasing values of ET was associated with an increased risk of HF, in patients with preserved LVEF. This phenomenon has previously been described in other studies [18, 35] and may be due to the worsening of LV systolic function, where myocardial myocyte’s ability to maintain high LV pressure decreases [8] resulting in the prolongation of time to achieve efficient pressure in the ventricles exceeding that of the aortic and pulmonary arteries, leading to prolongation of the IVCT and shortening of the ET. This in turn results in ineffective myocardial contraction, increasing LV wall thickness, and the development of HF [35].

### Perspective

Besides revealing myocardial dysfunction in patients with ischemic heart disease, which seems to be associated with cardiovascular events, the cardiac time intervals may also provide value for detecting patients who could benefit from targeted HF treatment. Recently, a selective cardiac myosin activator called Omecamtiv Mecarbil, was developed and tested in phase 2 and 3 trials [37, 38]. The GALACTIC-HF trial found that in HFrEF patients, Omecamtiv Mercabil significantly reduced the risk of HF events and cardiovascular death compared to placebo [39]. Prior to that, in the COSMIC-HF trial, Omecamtiv Mercabil was found to improve systolic function, in part through an increase in ET which resulting in favorable remodeling of the LV in patients with chronic HF with reduced ejection fraction [40]. This emphasizes that improvement of ET in turn translates into improved outcomes in HF patients and that ET may be used to identify patients at risk of HF and guide HF management.

### Limitations

This was a retrospective study, which is prone to residual confounding. The analysis, however, was made from a well-defined registry with systematic data collection. Differences were noted when comparing patients who were included vs. excluded in this study analysis, which suggests that some selection bias was present. Additionally, patients were enrolled from 2003 to 2008 and the patients may therefore not be representative of contemporary patients with ACS. Accordingly, our findings are primarily hypothesis-generating and should be validated both prospectively and in a contemporary study. The endpoint – incident HF - data were obtained from International Classifications of Diseases (ICD-10) codes from the Danish National Board of Health’s National Patient Registry and the Danish Register of Causes of Death may be questioned. However, the value of ICD-10 diagnostic codes for HF after reviewing medical records and discharge summaries has shown to have a positive predictive value of 100% [41].

## Conclusions

Decreasing ET and increasing MPI, assessed by curved M-mode tissue Doppler imaging, were associated with an increased risk of incident HF. Furthermore, lower values of ET were associated with incident HF in patients with LVEF above 36%. Accordingly, cardiac time intervals could assist in the risk stratification of HF after ACS.

## Electronic supplementary material

Below is the link to the electronic supplementary material.

Supplementary Material 1

## Data Availability

In compliance with Danish legislation, the data cannot be shared publicly as it contains sensitive patient information.
