# Supplementary material for: Association between cardiac time intervals and incident heart failure after acute coronary syndrome
Source: Int J Cardiovasc Imaging. 2024 Aug 3;40(10):2145–55. doi: 10.1007/s10554-024-03206-8 (PMC11499364; doi:10.1007/s10554-024-03206-8)
Supplement: Supplementary file 1 — Supplementary Material 1 [file 10554_2024_3206_MOESM1_ESM.docx]

**Supplementary Material**

Association between Cardiac Time Intervals and Incident Heart Failure after Acute Coronary Syndrome
Caroline Løkke Bjerregaard, MB^1,2,3^; Flemming Javier Olsen, MD, PhD^1,2,3^; Kristoffer Grundtvig Skaarup, MD^1,2,3^; Peter Godsk Jørgensen MD, PhD^1^; Søren Galatius, MD, DMSc^4^; Sune Pedersen MD, PhD^1^; Allan Iversen MD, PhD^1^; Tor Biering-Sørensen, MD, MSc, MPH, PhD^1,2,3,5,6^

1 Department of Cardiology, Copenhagen University Hospital - Herlev and Gentofte, Hellerup, Denmark.
2 Cardiovascular Non-Invasive Imaging Research Laboratory, Department of Cardiology, Herlev & Gentofte Hospital, University of Copenhagen, Denmark.
3 Center for Translational Cardiology and Pragmatic Randomized Trials, Department of Biomedical Sciences, Faculty of Health and Medical Sciences, University of Copenhagen.
4 Department of Cardiology, Copenhagen University Hospital – Bispebjerg and Frederiksberg, Copenhagen, Denmark
5 Department of Biomedical Sciences, Faculty of Health and Medical Sciences, University of Copenhagen, Copenhagen, Denmark.
6 Department of Cardiology, Copenhagen University Hospital – Rigshospitalet, Copenhagen, Denmark

Word count: 2827 (excluding title page, abstract, references, tables, figure legends)

Abstract word count: 250

Running title: Cardiac time intervals in acute MI

**Correspondence:**

Caroline Løkke Bjerregaard
Center for Translational Cardiology and Pragmatic Randomized Trials, Department of Biomedical Sciences, Faculty of Health and Medical Sciences, University of Copenhagen.

Cardiovascular Non-Invasive Imaging Research Laboratory, Department of Cardiology, Herlev & Gentofte Hospital, University of Copenhagen, Denmark
Niels Andersens Vej 65, 2900 Hellerup, Denmark
Phone: +45 50 45 86 18 ; Fax: +45 39 77 73 81
E-mail: [bjerregaard.caroline@gmail.com](mailto:bjerregaard.caroline@gmail.com)

**Supplementary Table 1 – Included vs. excluded eligible patients**

| Variable | Included in analysis | Excluded from analysis | | P-value |
| --- | --- | --- | --- | --- |
|  | (n: 386) | (n: 194) |  |  |
| **Clinical characteristics** |  |  | |  |
| Age, years | 64 ± 12 | 66 ± 13 | | 0.048 |
| Male gender, % | 73 | 73 | | 0.50 |
| Heart rate, beats per minute | 73 ± 14 | 81 ± 19 | | <0.001 |
| Body mass index, kg/m^2^ | 26 ± 4 | 27 ± 7 | | 0.28 |
| Systolic blood pressure, mmHg | 138 ± 25 | 128 ± 30 | | <0.001 |
| Diastolic blood pressure, mmHg | 82 ± 16 | 76 ± 82 | | <0.001 |
| Mean arterial pressure, mmHg | 119 ± 21 | 110 ± 25 | | <0.001 |
| Diabetes mellitus, % | 9 | 12 | | 0.23 |
| Current smokers, % | 48 | 39 | | 0.042 |
| Hypercholesterolemia, % | 23 | 28 | | 0.15 |
| Family history of CV disease, % | 31 | 28 | | 0.53 |
| Prior CV disease, % | 8 | 13 | | 0.10 |
| **Hospitalization** |  |  | |  |
| *Diagnosis* |  |  | |  |
| STEMI, % | 76 | 75 | | 0.84 |
| NSTEMI and/or UAP, % | 24 | 25 | | 0.84 |
| *Culprit lesion* |  |  | | 0.015 |
| Left circumflex artery, % | 14 | 14 | |  |
| Left anterior descending artery, % | 51 | 47 | |  |
| Right coronary artery, % | 36 | 37 | |  |
| Left main stem, % | 0 | 3 | |  |
| Multivessel disease, % | 6 | 7 | | 0.69 |

*Continuous variables with Gaussian distribution are shown as mean ± standard deviations, skewed variables as median with interquartile range, and proportion as total numbers and percentages.
Abbreviations: CV: Cardiovascular; LV: Left ventricular; STEMI: ST-elevation myocardial infarction; NSTEMI: Non-ST-elevation myocardial infarction; UAP: Unstable angina pectoris*

**Supplementary Table 2**

Baseline characteristics stratified for the entire study population and according to heart failure.

| Factor | No heart failure | Heart failure | P-value |
| --- | --- | --- | --- |
|  | (n=246) | (n=140) |  |
| **Clinical characteristics** |  |  |  |
| Age, years | 64 ± 12 | 65 ± 12 | 0.41 |
| Male gender, n (%) | 178 (72) | 105 (75) | 0.57 |
| Heart rate, beats per minute | 71 ± 13 | 77 ± 15 | <0.001 |
| Body mass index, kg/m^2^ | 26 ± 4 | 27 ± 5 | 0.31 |
| Systolic blood pressure, mmHg | 139 ± 24 | 138 ± 27 | 0.82 |
| Diastolic blood pressure, mmHg | 82 ± 16 | 81 ± 16 | 0.40 |
| Mean arterial pressure, mmHg | 120 ± 20 | 119 ± 22 | 0.69 |
| Diabetes mellitus, n (%) | 20 (8) | 14 (10) | 0.53 |
| Current smokers, n (%) | 113 (46) | 71 (51) | 0.37 |
| Hypercholesterolemia, n (%) | 56 (23) | 32 (23) | 0.98 |
| Family history of CV disease, n (%) | 81 (33) | 38 (27) | 0.24 |
| Prior CV disease, n (%) | 14 (6) | 18 (13) | 0.014 |
| **Hospitalization** |  |  |  |
| *Diagnosis* |  |  |  |
| STEMI, n (%) | 182 (74) | 112 (80) | 0.18 |
| NSTEMI and/or UAP, n (%) | 64 (26) | 28 (20) | 0.18 |
| *Culprit lesion* |  |  | <0.001 |
| Left circumflex artery, n (%) | 40 (16) | 12 (9) |  |
| Left anterior descending artery, n (%) | 107 (44) | 88 (63) |  |
| Right coronary artery, n (%) | 99 (40) | 40 (29) |  |
| Multivessel disease, n (%) | 18 (7) | 5 (4) | 0.14 |
| **Echocardiography** |  |  |  |
| Interventricular septal thickness, cm | 1.1 ± 0.2 | 1.1 ± 0.2 | 0.85 |
| LV internal diameter, cm | 5.0 ± 0.5 | 5.0 ± 0.6 | 0.043 |
| LV posterior wall thickness, cm | 0.9 ± 0.2 | 1.0 ± 0.2 | 0.007 |
| Left ventricular mass index, g/m^2^ | 95 ± 19 | 97 ± 30 | 0.64 |
| Left ventricular ejection fraction, % | 44 ± 10 | 37 ± 11 | <0.001 |
| E/A ratio | 0.99 [0.80-1.25] | 0.95 [0.78-1.22] | 0.25 |
| E/e’ ratio | 9.4 [7.7-11.7] | 9.9 [8.1-14.2] | 0.014 |
| e’, cm/s | 7.7 ± 2.3 | 6.8 ±2.1 | <0.001 |
| Deceleration time, ms | 177 ± 44 | 165 ± 49 | 0.023 |
| Left atrial volume index, mL/m^2^ | 30.3 ± 10.7 | 27.8 ± 10.5 | 0.23 |
| TAPSE, cm | 1.9 ± 0.4 | 1.8 ± 0.4 | 0.036 |
| Global longitudinal strain, % | -14.4 ± 3.4 | - 11.0 ± 3.2 | <0.001 |
| *Cardiac time intervals* |  |  |  |
| IVRT, ms | 99 ± 22 | 100 ± 26 | 0.47 |
| IVCT, ms | 27 [19-36] | 28 [17-38] | 0.76 |
| ET, ms | 267 [245-285] | 248 [230-267] | <0.001 |
| MPI | 0.48 [0.40-0.55] | 0.53 [0.42-0.62] | 0.007 |

*Continuous variables with Gaussian distribution are shown as mean ± standard deviations, skewed variables as median with interquartile range, and proportion as total numbers and percentages.
Abbreviations: CV: Cardiovascular; LV: Left ventricular; STEMI: ST-elevation myocardial infarction; NSTEMI: Non-ST-elevation myocardial infarction; UAP: Unstable angina pectoris; TAPSE: Tricuspid annular plane systolic excursion; IVRT: Isovolumic relaxation time; IVCT: Isovolumic contraction time, ET: systolic ejection time; MPI: Myocardial performance index*
